# Innate immune evasion by alphaviruses

**DOI:** 10.3389/fimmu.2022.1005586

**Published:** 2022-09-12

**Authors:** Yihan Liu, Yupei Yuan, Leiliang Zhang

**Affiliations:** ^1^Department of Infectious Diseases, Shandong Provincial Hospital Affiliated to Shandong First Medical University, Jinan, China; ^2^Department of Pathogen Biology, School of Clinical and Basic Medical Sciences, Shandong First Medical University & Shandong Academy of Medical Sciences, Jinan, China; ^3^Medical Science and Technology Innovation Center, Shandong First Medical University & Shandong Academy of Medical Sciences, Jinan, China

**Keywords:** innate immune evasion, alphavirus, CHIKV, interferon, STING

## Abstract

Alphaviruses contain many human and animal pathogens, such as CHIKV, SINV, and VEEV. Accumulating evidence indicates that innate immunity plays an important role in response to alphaviruses infection. In parallel, alphaviruses have evolved many strategies to evade host antiviral innate immunity. In the current review, we focus on the underlying mechanisms employed by alphaviruses to evade cGAS-STING, IFN, transcriptional host shutoff, translational host shutoff, and RNAi. Dissecting the detailed antiviral immune evasion mechanisms by alphaviruses will enhance our understanding of the pathogenesis of alphaviruses and may provide more effective strategies to control alphaviruses infection.

## Introduction

Alphaviruses are positive-stranded RNA viruses and belong to the *Togaviridae* family (1). It contains many important human and animal pathogens, such as chikungunya virus (CHIKV), Sindbis virus (SINV), and Venezuelan equine encephalitis virus (VEEV). Affected by geographical factors and climatic conditions, alphaviruses distribute on all continents except Antarctica and many islands. CHIKV can be found in tropical and subtropical regions of Africa and Southeast Asia, where winter temperatures are above 18°C. This virus is famous for causing Chikungunya fever, with the symptoms of acute febrile illness, arthralgia, and severe neurological complications (2).SINV exists in Europe, Asia, and Africa, including many Philippine Islands and the South Pacific areas. Fever, malaise, rash, and chronic musculoskeletal pain are the main symptoms of SINV (3). VEEV is mainly in circulation in the American continent and can cause severe encephalitis (4). The major transmission vectors of alphaviruses are mosquitoes, including *Aedes aegypti*, *Aedes albopictus*, and *Aedes africanus* (5).

Alphavirus particles are round with a diameter of about 70 nanometers. The viral nucleocapsid has an icosahedral symmetry with a diameter of 30-40 nanometers. Alphavirus genome comprises a 5’-methylguanylate cap, a 3’-polyadenylic acid tail, and two open reading frames (ORFs), which encode four nonstructural proteins and six structural proteins (6). Nonstructural proteins include nsP1, nsP2, nsP3 and nsP4, and they play critical roles in the transcription and replication of the virus (7). nsP1 is required for cap synthesis and plasma membrane-anchoring. nsP2 is necessary for polyproteins processing as its C terminus obtains an N-terminal RNA helicase and cysteine protease (8). ADP ribosyl-binding and hydrolase activities in nsP3 are crucial for viral replication. nsP4 activity depends on its RNA polymerase activity. Structural proteins of alphaviruses are capsid, 6K, transferase protein (TF), E1, E2, and E3 (7). The capsid protein is used for packaging the viral nucleic acid (7). 6K participates in the infected cell surface’s vial assembly and budding stages. Shared with the same coding regions with 6K, TF is generated due to a ribosomal frameshift and promotes virus replication by reducing the early IFN-I response (9). E1 and E2 mediate the entry of the virus. 6K and E3 work together to transport the precursor membrane protein to the endoplasmic reticulum (ER) (7).

The host innate immune system is the first line of defense against viral infection. For example, the Cyclic GMP-AMP Synthase (cGAS)-stimulator of interferon genes (STING) pathway could stimulate and promote the production of type I interferon to achieve antiviral effects (10). Degradation of the key regulator of eukaryotic messenger RNA transcription, RPB1, will induce transcriptional host shutoff (11). Signal transduction of the PKR-like ER kinases (PERK) pathway and the unfolded protein response (UPR) phosphorylates eukaryotic translation initiation factor 2 (eIF2), which causes translational shutoff (11). RNA-induced silencing complex (RISC) can cleave viral RNA and activate RNAi (12).

However, with the evolution of viruses and their long-term confrontation with host cells, many viruses have established effective antagonisms to host antiviral innate immune pathways and immune factors (13–21). This review has summarized different mechanisms of how alphaviruses antagonize the host’s innate immunity. Alphavirus is highly infectious as it can transmit by the mosquito and pose a huge threat to public health. So understanding the antiviral innate immune pathway and the antagonistic effects induced by the viral proteins of alphaviruses could provide more strategies to control the diseases caused by alphaviruses.

## Restraint of cGAS-STING pathway

When infected by a virus, activation of cytoplasmic DNA sensors such as cGAS in immune cells is adapted to intracellular damage caused by the released viral DNA. The 2’-3’ cyclic-guanosine monophosphate (GMP)-adenosine monophosphate (AMP) (GAMP) is synthesized to bind to STING, which then forms dimerization and translocates to the Trans-Golgi-Network (TGN) and associate with TANK-Binding Kinase 1 (TBK1), resulting in the phosphorylation of Interferon Regulatory Factor 3 (IRF3) (22). The transcription and expression of the cGAS-STING innate immune pathway could inhibit virus replication, whereas viruses could antagonize this process.

**Figure 1:**
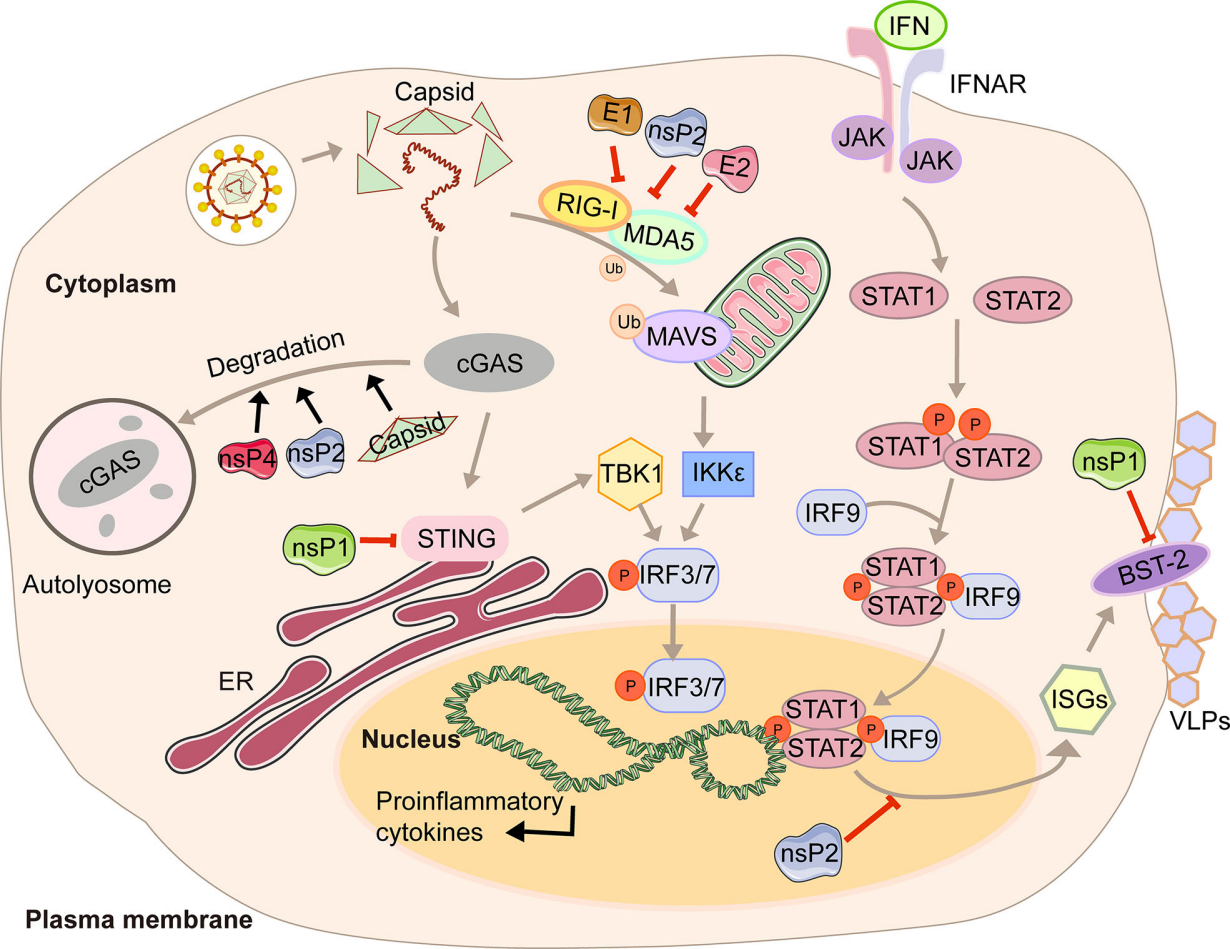
A diagram of alphavirus-mediated inhibition of the innate immune pathways leading to the production of IFN and ISG induction.

In the first four hours of CHIKV infection, the expression of cGAS is reduced sharply, while there is no significant change in the expression of STING (23). The degradation of cGAS is mediated by capsid protein through ATG7-dependent autophagy (**Figure 1**). When the chemical inhibitor of autophagy 3-methyladenine (3-MA) is used, cGAS can be restored. Capsid-mediated cGAS degradation directly limits the antiviral effect of the cGAS-STING pathway (23). The interaction between nsP1 and STING is mediated by the cytoplasmic loop of STING, mainly due to the palmitoylation that occurs at 88 and 91 amino acids. The level of viral protein will be significantly reduced, and the cGAS-STING-mediated induction of type I IFN will be impaired when this interaction is lost (23). Interestingly, nsP1-STING interaction significantly inhibits IFNβ promotor activation induced by cGAS-STING (23). Other viruses degrade components of the cGAS-STING signaling to achieve evasion. For example, DENV NS2B3 protease inhibits type I IFN production in infected cells by cleaving STING (23), and papain-like protease (PLpro)-transmembrane domain (TM) in SARS disrupts IRF3 phosphorylation and dimerization (24). Interestingly, nsP2, nsP4, and capsid proteins have separate or synergistic effects on the degradation of cGAS, and their mechanisms are worthy of further study and discussion.

## Inhibition of IFN pathway

Type I interferon (IFN) is a cytokine is crucial for the antiviral response and activation of the innate and adaptive immune system. Its production is often triggered by pattern recognition receptors (PRRs), including Toll-like receptors (TLRs), retinoic acid-inducible gene I (RIG-I)-like receptors (RIG-I-like receptors, RLRs), nucleotide-binding and oligomerization domain (NOD)-like receptors (NLRs), and intracellular DNA receptors (25, 26). After the cytoplasmic receptor recognizes the viral nucleic acid, melanoma differentiation-associated gene 5 (MDA5) and RIG-I will expose the caspase recruitment domain (CARD) domain to induce the aggregation and activation of the mitochondrial antiviral signal protein (MAVS). Then the signal is transmitted downwards to activate TBK1 and IKKϵso that IRF3/7 is phosphorylated and transported to the nucleus to promote the transcription of type I interferons (27). The viral genome contains two ORFs. The first encodes precursor proteins, and the second encodes structural polyproteins, where the 6K protein causes a ribosomal frameshifting during translation and produces the TF (9). Virus modification of host protein often occurs through post-translational modifications, such as palmitoylation and phosphorylation. Palmitoylation is a 16-carbon palmitoyl covalent bond attached to cysteine residues. This modification imparts hydrophobicity to the protein and is usually targeted at the cell membrane (28). TF has been demonstrated to be modified by palmitoylation (29, 30). 6K mutation indicates that hexanucleotide cannot be reduced to produce TF protein (31, 32). The loss of TF palmitoylation will result in the enrichment of the type I IFN production and the attenuation of toxicity caused by SINV infection (33). Palmitoylated TF is necessary for its localization and the subsequent production of virus particles, which also helps to enhance the ability to antagonize the host interferon response. Meanwhile, nsP2, E1, and E2 proteins of CHIKV can strongly antagonize the activation of the IFN-β signaling pathway (**Figure 1**). Co-expression of nsP2, E1, E2, and MDA5/RIG-I allows the inhibition of more than 80% of the MDA5/RIG-I-mediated IFN-β promoter activity in the presence of viral proteins (34).

In response to IFN, IFN receptors phosphorylate signal transducer and activator of transcription 1 (STAT1) (35). Then the importin-α5 transports the phosphorylation form of STAT1 (pSTAT1) to the nucleus, together with IFN response factor 9 (IRF9), and binds to the IFN-stimulated response element (ISRE) so that the transcription of the IFN-stimulated genes (ISGs) is activated (35). With the help of chromosome region maintenance 1 (CRM1), STAT1 is shuttled back into the cytoplasm when achieving the goal of releasing from its target promoter (35). This signaling pathway restricts CHIKV propagation and abolishes CHIKV-induced diseases (36). However, CHIKV infection effectively inhibits IFN-mediated phosphorylation of STAT1, thereby hindering the transmission of JAK-STAT immune signals (36). CHIKV nsP2 is responsible for regulating the IFN-induced JAK-STAT signaling (**Figure 1**) (37). By mutating the KR649AA site in NLS of nsP2 or redirecting nsP2 C-terminal methyltransferase-like domain into the nucleus, JAK-STAT signaling is no longer inhibited mechanically due to the reduction of pSTAT nuclear accumulation (38, 39).

Antiviral effects of IFN are fulfilled by antiviral IFN-stimulate genes (ISGs). One well-characterized ISG is bone marrow stromal antigen 2 (BST-2). Its transmembrane domain and lumenal GPI anchor allow virus particles to adhere to the surface of infected cells, thereby preventing release and bystander cells’ infection (40). Although BST-2 could block the release of the virus, many of which have evolved multiple mechanisms to antagonize the inhibitory effect (40, 41), which is also the case for CHIKV. CHIKV protein co-localizes with BST-2 when expressed in VLPs, namely E1 and nsP1 (42). There are interactions between BST-2 and E1 and nsP1, but the protein that antagonizes its inhibitory effect on virus release is nsP1. In the presence of BST-2, a CHIKV virus-like particle (VLP) is adhered to the cell membrane and cannot be released from the surface. However, acting as an antagonist, upregulation of nsP1 counteracts the effect of BST-2, which enables the progeny virions to attach to the membrane (**Figure 1**) (42). The same effects can be observed in HIV-1 Vpu and HIV-2 Env, which can redirect the BST-2 from the cell surface and form a perinuclear compartment (41, 43). The prerequisite is that Vpu must have both transmembrane/ion channel domain and conserved proteins (40).

In addition, the alphavirus can not only use its viral protein to inhibit the production, translation, and transcription of interferon but also use the host antiviral protein to achieve immune escape, such as zinc-finger antiviral protein (ZAP). ZAP is a host antiviral factor stimulated by IFN, inhibiting the replication of some viruses, including HBV, Sindbis virus, and Ebola (44–46). Due to the interaction between ZAP-responsible elements (ZRE) and viral RNA, some exosomes are recruited to degrade RNA substrates (44, 47). Sometimes ZAP could disturb the polysome association/translation of RNA (45). In ZAP gene knockout mice models, virus replications are greatly enhanced in lymphoid tissues, while this phenomenon could not be observed in brain tissues. Those results imply that viral infection can evade immune surveillance by suppressing the expression of ZAP antiviral protein in the brain tissues (47). However, there may be other ways for the virus to achieve antiviral effects and immune escape in the host, which requires further research.

## Suppression of transcriptional host shutoff pathway

A basic feature of massive alphavirus replication in vertebrates is the cytopathic effect (CPE). Alphavirus inhibits the occurrence and efficacy of the host antiviral response by antagonizing cell transcription so that it can replicate *in vivo*, which is achieved through different mechanisms mediated by alphavirus proteins.

**Figure 2:**
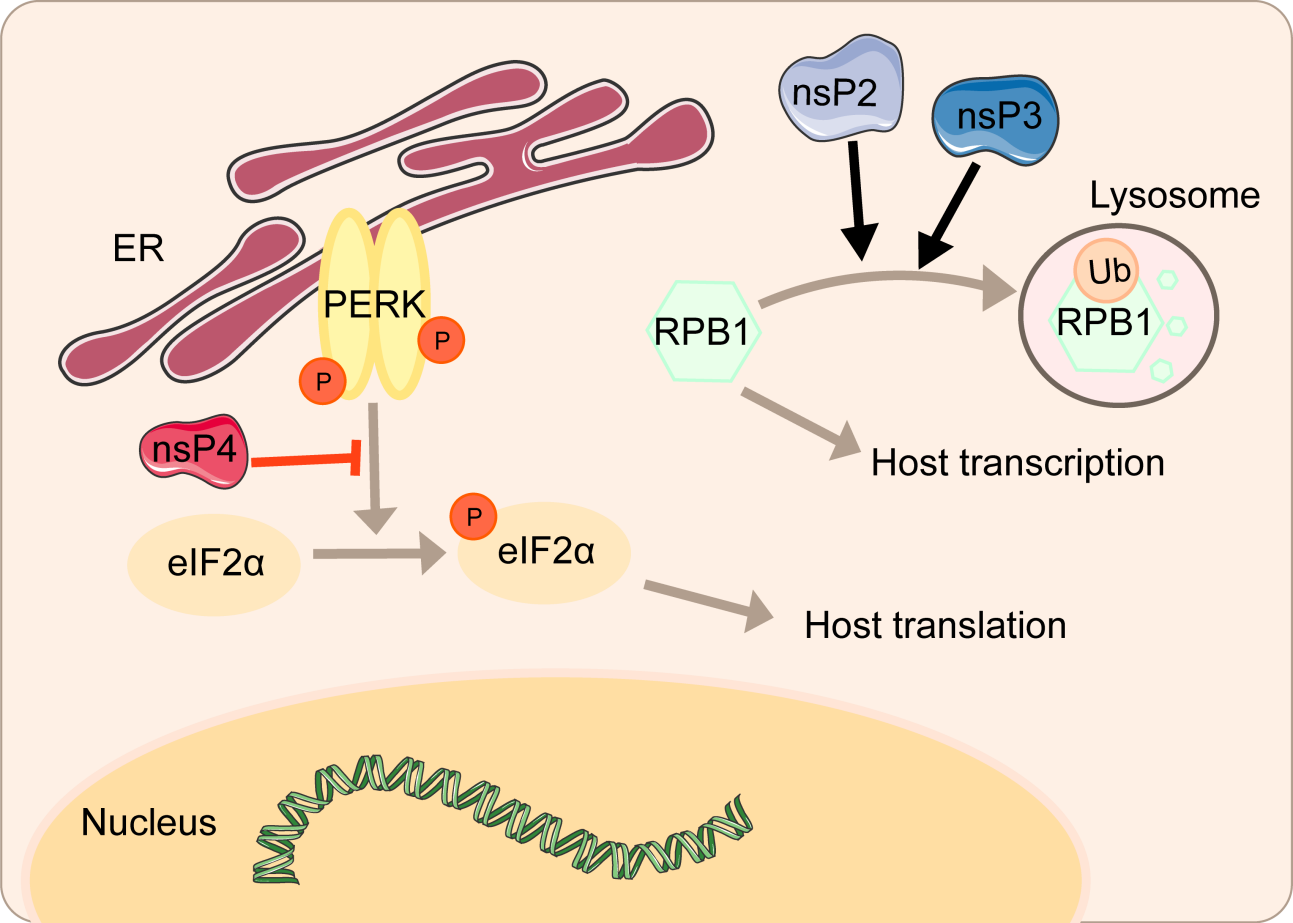
Inhibition of host transcriptional and translational shutoff by alphaviruses.

nsP2 from old world alphaviruses, including SINV and SFV, is the key regulator of the interaction between the virus and the host cell. Not only does nsP2 serves as a component of the replicase complex required for viral RNA replication and transcription, but also it can directly participate in the inhibition of host transcription (48, 49). As a subunit that catalyzes the polymerase reaction, RPB1 determines the initiation and extension of eukaryotic messenger RNA transcription. nsP2 could induce the ubiquitination and degradation of RPB1 (**Figure 2**) (50). In the experiment of mice infected with SINV containing a single nsP2 substitution (P726→G), a significant increase in the secretion of IFN can be seen due to the shutdown of host transcription (50). Normally, cells can remove the extended RNAPII complex during the transcription-coupled repair. Once the complex is blocked by large amounts of DNA damage, RPB1 can be modified by ubiquitination and then degraded by the proteasome (51). Mutating amino acids 674-688 can resist virus-induced degradation of RPB1 and make SINV a powerful inducer of type I interferon (48). It is worth noting that this phenomenon does not affect virus replication.

The amino terminal region of alphavirus nsP3 has the effect of a single ADP-ribosylhydrolase, and the N24A mutation in this region eliminates the hydrolase activity (52). A single mutation in N24A still induces the degradation of RPB1, while the double mutation of SINV nsP2-683S and nsP3-N24A no longer degrades RPB1 (48). Mayaro virus (MAYV) nsP2 associates with RPB1 and transcription initiation factor IIE subunit 2 (TFIIE2) (53). Overexpression of MAYV nsP2 mediates inhibition of host cell transcription by reducing RPB1 and TFIIE2 (53).

The cytotoxicity of the new world alphaviruses represented by VEEV and EEEV differs from that of the old world alphaviruses. VEEV and EEEV-derived replicons produce fewer cytopathic changes, and at the same time, durable viral nucleic acid replication can be established (54). This host transcription shutoff in VEEV and EEEV depends on the presence of viral capsid protein (55). The capsid is distributed in the cytoplasm of infected cells and may interfere with the antiviral response. Capsid could inhibit cell messenger and ribosomal RNA transcription and downregulate RNA synthesis. Interestingly, western equine encephalitis virus (WEEV) inhibits the host transcription depending on both nsP2 and capsid, consisting of the current concept of forming WEEV from SINV- and EEEV-like ancestors (55).

## Suppression of translational host shutoff pathway

After mammals are infected with alphavirus, the replication of viral RNA in the cell often leads to more serious cytopathic changes. That is, selective inhibition of host protein synthesis and viral mRNA will be in this case. The ER is responsible for the proper folding and processing of polypeptide chains into functional proteins. Factors affecting the function of the ER, such as viral infection, will lead to the accumulation of misfolded or unfolded proteins. To protect cells from over accumulation, repression of protein synthesis, so-called (UPR), maintains cellular protein homeostasis (56). These regulatory signalings contain (PERK), transcription factor 6 (ATF6), and the ER transmembrane protein kinase/endoribonuclease inositol-requiring enzyme 1 (IRE1), with the involvement of ER chaperone immunoglobulin heavy chain binding protein (BIP) (56). PERK can be activated through self-dimerization and phosphorylation, then pPERK can phosphorylate eIF2α on amino acid 51, during which GADD34 can play an inhibitory role against this process (57). Induction of C/EBP homologous protein (CHOP) is to mediate apoptosis when ER is impaired severely. The IRE pathway is activated similarly. The catalytic of IRE will trigger a sequence of gene transcription, such as components of ER-associated degradation (ERAD). ATF6 activates transcription of the chaperone, thus helping translational recovery (58, 59).

However, the virus can regulate the activity of some key factors to influence the antagonism of protein synthesis, ensuring the effective translation of virus mRNAs and the shutoff of host translation. CHIKV can regulate the signal transduction of the PERK pathway by inhibiting the phosphorylation of eIF2α during early infection (**Figure 2**). Upon significant expression of CHIKV nsP4, the phosphorylation of eIF2α on serine 51 regulating the signal transduction of PERK is suppressed, thereby ensuring the translation of viral proteins (58). Overexpression of CHIKV nsP2 inhibits the expression of functional UPR transcription factors ATF4 and activation of XBP1 and thus blocks the UPR, which is another strategy to shut off host translation (60).

## Inhibition of RNAi pathway

Eukaryotes have evolved many antiviral immune defenses to prevent viral infection, such as RNA interference(RNAi) (12). RNAi is a conservative post-transcriptional gene silencing mechanism (12). As a member of the RNase III family of nucleases, Dicer has a helicase domain and dual RNase III motif, and it can cleave double-stranded RNAs specifically. In the process of antiviral RNAi, host cells respond rapidly to the dsRNA derived from the invading viruses, activating Dicer to cleave the dsRNA into virus-derived small interfering RNA (viRNA) with a size of 21 to 23 nucleotides, which is crucial for the antiviral response (12). One of the components of the RISC, Argonaute (AGO), plays an indispensable role in degrading the target dsRNA achieved through the RNase-H-like PiWi domain or recruiting additional proteins, thereby inhibiting viral infection (12). However, protection against RNAi attack enables the virus to encode specific virulence proteins, the so-called viral suppressors of RNAi (VSRs) (12).

**Figure 3:**
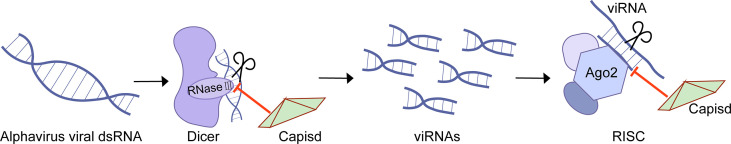
Inhibition of RNAi by alphaviruses.

The Semliki forest virus (SFV) capsid protein is demonstrated as VSR (61). SFV capsid protects viral RNA from interpretation at two stages. On the one hand, capsid binds to dsRNA to block Dicer cleavage and thus antagonizes the production of viRNA (**Figure 3**). The experiments of SFV capsid mutants suggest that K124/K128 and K139/K142 are essential for VSR activity (61). On the other hand, capsid associates with viRNA and thus prevents the interaction between viRNA and RISC (61). Consequently, the inactivation of the VSR function will inhibit SFV replication.

## Conclusion and perspectives

In this review, we have described in detail the different mechanisms by which each viral protein of alphavirus antagonizes the host’s innate immunity. The host will recognize virus invasion through the sensor proteins, including cGAS and MAVS, and activate the antiviral innate immune pathway. The downstream signals further activate the TBK1-IRF3 and IKK-NF-κB pathways, increasing type I interferon production and inhibiting viral infection. Almost all alphavirus proteins antagonize innate immunity in different mechanisms and degrees. Through the interaction with cGAS-STING, nsP1 degrades cGAS to stabilize the virus protein (**Figure 1**). nsP1 also down-regulates the expression of BST-2 to inhibit the adhesion of VLPs on the plasma membrane and promote the release of the virus. The mechanism of nsP2 antagonizing immune response is more complex. The type I interferon response can be counteracted by inhibiting the general transcription of host cells and reducing the phosphorylation of STAT1 in the JAK-STAT pathway (**Figure 1**). In addition, degradation of RPB1 occurs by nsP2-mediated ubiquitination, through which the transcription of the host proteins can be shut down (**Figure 2**). At the same time, nsP2 exerts a profound impact on the phosphorylation of STAT1 and STAT2 and thus inhibits the host translation (**Figure 1**). Both nsP3 and nsP4 could induce the host transcriptional shutdown (**Figure 2**). NsP4 inhibits the phosphorylation of eIF2alpha in the PERK pathway. Among the structural proteins, the capsid protein can effectively inhibit RNAi in insect and mammalian cells by separating double-stranded RNA and small interfering RNA (**Figure 3**). E2 and E1 inhibit the activation of the IFN-β promoter induced by the MDA5/RIG-I receptor signaling pathway. TF antagonizes the host interferon response. In addition to the structural and nonstructural proteins of alphavirus, the virus also uses the immune escape phenomenon of ZAP to antagonize the host’s antiviral response.

As more and more studies are performed, a deeper and more comprehensive understanding of alphavirus antagonizing host antiviral innate immunity is revealed. However, some mechanisms are not clear enough, and there may be other ways and mechanisms to antagonize antiviral immunity that are worthy of further research and exploration. At the same time, the strategies of antagonizing antiviral immunity by alphaviruses will provide important insights into controlling viral infections.

## Author contributions

LZ conceived the work. YL and YY wrote the draft. YL generated Figures. LZ revised the manuscript. All approved the final version for publication.

## Funding

This work was supported by grants from the National Natural Science Foundation of China [81871663 and 82072270] and the Academic Promotion Program of Shandong First Medical University [2019LJ001].

## Conflict of interest

The authors declare that the research was conducted in the absence of any commercial or financial relationships that could be construed as a potential conflict of interest.

## Publisher’s note

All claims expressed in this article are solely those of the authors and do not necessarily represent those of their affiliated organizations, or those of the publisher, the editors and the reviewers. Any product that may be evaluated in this article, or claim that may be made by its manufacturer, is not guaranteed or endorsed by the publisher.
